# Pneumococcus nasopharyngeal carriage in children attending an academic hospital in Pretoria, South Africa, after the introduction of pneumococcal vaccine

**DOI:** 10.4102/sajid.v34i1.112

**Published:** 2019-10-22

**Authors:** Charity Newton, Harry Maake, Caroline Maluleka, Siyazi Mda

**Affiliations:** 1 Department of Paediatrics and Child Health, Faculty of Health Sciences, Sefako Makgatho Health Sciences University, Pretoria, South Africa; 2 Department of Microbiology, Faculty of Health Sciences, Sefako Makgatho Health Sciences University, Pretoria, South Africa; 3 Faculty of Health Sciences, Nelson Mandela University, Port Elizabeth, South Africa

**Keywords:** Children, pneumococcal nasopharyngeal colonisation, immunisation, serotypes, serotype replacement

## Abstract

**Background:**

In 2009, pneumococcal conjugate vaccine was introduced in South Africa. However, there are concerns that this could lead to an increase in colonisation of non-vaccine serotypes (serotype replacement).

**Methods:**

In a cross-sectional study, 350 children aged 1 month to 14 years were enrolled at Dr George Mukhari Academic Hospital from December 2015 to April 2016. We assessed the prevalence of nasopharyngeal colonisation with pneumococcus and characterised the serotypes found.

**Results:**

The median age of the cohort was 33.7 months (interquartile range 16.27–69.5 months), with 20% being < 1 year. A total of 21% of the children were diagnosed with pneumococcal-related conditions; among these, pneumonia was the most common condition. Less than half (43%) of the participants were fully immunised. Forty-six (13%) of the children were colonised with pneumococcus. Younger age was significantly associated with pneumococcal colonisation. Among those colonised with pneumococcus, 35% were fully immunised, 30% were partially immunised, 30% had an unknown immunisation status and 4% were unimmunised. Eight (17%) of the children who were colonised with pneumococcus had pneumococcal-related conditions. The commonest serotype identified was 6A/B. Overall, 2% of the cohort were colonised with vaccine-serotype pneumococcus.

**Conclusion:**

As a minority of children had evidence of nasopharyngeal colonisation with vaccine-serotype pneumococci, serotype replacement may be emerging in our population.

## Introduction

It is estimated that pneumococcal disease accounts for about 300 000 deaths in children, and half of these occur in Africa.^1^ The incidence of pneumococcal disease differs in many populations and factors such as overcrowding, poverty, human immunodeficiency virus (HIV) and genetics have been attributed as contributing to these differences.^2^

*Streptococcus pneumoniae* (pneumococcus) is part of the commensal flora of the respiratory tract.^3,4^ Nasopharyngeal pneumococcal colonisation is common in children^5^ and it is a prerequisite for developing pneumococcal disease.^2,4,6^ Colonisation is the main source for transmission of the bacterium within communities; therefore, preventing nasopharyngeal colonisation in children is a strategy for preventing disease in children and adults.^4^ Immunisation of children with pneumococcal conjugate vaccine (PCV) has been shown to decrease the risk of acquiring vaccine serotype nasopharyngeal colonisation.^7^

There are more than 90 pneumococcal serotypes;^8,9^ however, before the introduction of PCV, about 10 serotypes accounted for more than 70% of invasive pneumococcal disease (IPD) in children globally.^9^ Invasive pneumococcal disease is defined as isolation of pneumococcus from a normally sterile site, for example, cerebrospinal fluid, blood, joint fluid and pleural fluid.^9,10,11^ It includes pneumonia, septicaemia and meningitis caused by pneumococcus.^9^

In 2009, South Africa introduced PCV into its routine immunisation programme.^12^ The 7-valent PCV (PCV7) that targets serotypes 4, 6B, 9V, 14, 18C, 19F and 23F^11^ was introduced with two primary doses, given to infants at 6 and 14 weeks of age and a booster at 9 months.^13^ In 2011, a 13-valent PCV (PCV13) replaced PCV7.^14^ In addition to serotypes contained in PCV7, PCV13 includes serotypes 1, 3, 5, 6A, 7F and 19A.^11,15^ Surveillance among South African children from 2005 to 2012 showed that the introduction of PCV7 had been effective in reducing the rate of PCV7 serotype IPD.^13^

Some studies in Europe and the United States after the introduction of PCV have shown an increase in the prevalence of carriage of serotypes not included in the vaccine (non-vaccine serotypes [NVTs]), a phenomenon called serotype replacement.^16,17^ Similarly, in South Africa there has been an increase in NVT colonisation, although there has been no significant increase in NVT IPD.^18^

It is essential to monitor the pneumococcal colonisation prevalence in South African children subsequent to PCV introduction, and to characterise the colonising serotypes. The current study was designed to assess nasopharyngeal colonisation prevalence in South African children subsequent to PCV introduction, and to characterise the serotypes.

## Methods

In a cross-sectional descriptive study, children aged 1 month to 14 years attending the general outpatients department at Dr George Mukhari Academic Hospital in Pretoria, South Africa, were sequentially enrolled. Sample size calculations were performed using the prevalence formula *n* = *Z*²*P* (1-*P*)/*d*², where *Z* = 1.96 for confidence, *P* = expected prevalence and *d* = 0.05.^19^ Prevalence rates of 17% and 67% have been reported among Kenyan and South African study populations, respectively;^20,21^ these yielded sample sizes of 217 and 340, respectively. It was decided to enrol 350 children.

Exclusion criteria for the study included the following: children presenting with severe respiratory distress were excluded from the study.

The following information was documented: date of birth (used to calculate age), sex and clinical diagnosis. Children with meningitis, pneumonia, otitis media or tonsillitis were designated as having pneumococcal-related conditions.

Participant anthropometry (weight, length and/or height) was measured by the principal investigator with the help of an assistant. Each child’s weight was measured (approximated to the nearest 0.1 kg), using a single calibrated scale, with the child only wearing a clean nappy or underwear. Length was measured using a measuring board with a fixed upper part and a moveable lower part as per validated procedures.^22^ Height was measured with the child standing upright against a stadiometer, according to standardised procedures.^22^ Length and height were approximated to the nearest 0.1 cm. The age, weight and length or height were used to calculate weight-for-age Z-scores, weight-for-height Z-scores (WHZ) and height-for-age Z-scores (HAZ) using Epi-info software.

Participant HIV status was assigned by abstracting the result from clinical records. If an HIV result could not be obtained, the HIV status of the child was regarded as unknown.

The immunisation status of each child was obtained from their Road-to-Health card. A fully immunised child was described as a child who had received PCV doses at 6 weeks, 14 weeks and 9 months of age. A child who had not received all immunisations (including the child who was too young to have received all immunisations) was described as partially immunised and, if the child had not received any immunisations, the child was described as unimmunised. If the Road-to-Health card was not available, immunisation status was recorded as unknown.

Nasopharyngeal swabs were collected from all enrolled children by the principal investigator, according to World Health Organization-recommended standards^23^ and dispatched in Amies transport media for culture to the local National Health Laboratory Service laboratory within 3 h of collection. Swabs were inoculated onto 5% sheep blood agar plates (Diagnostic Media Products, Johannesburg, South Africa) and incubated at 37°C for 18–24 h.

*Streptococcus pneumoniae* was confirmed by appearance of colony morphology, gram stain, alpha haemolysis, optochin susceptibility and bile solubility testing. *Streptococcus pneumoniae* strain ATCC 49619 was used as a positive control, and the growth of *Streptococcus mitis* strain ATCC 49456 was used as a negative control.

Preserved pneumococcal isolates were used for DNA extraction using the ZR Genomic DNA-Tissue MiniPrep kit (Zymo Research Corporation, Irvine, CA, USA). Extracted DNA was stored at –20°C and used as a template for polymerase chain reaction (PCR). The DNA extracted was quantified using a NanoDrop Lite Spectrophotometer (Thermo Scientific, Waltham, MA, US) with a purity of above 2 (A260/280). Primers were grouped into nine multiplex reactions, with four primer pairs targeting serotype-specific sequences of the four different serotypes in each reaction. We were able to detect the following vaccine serotypes: 1, 3, 4, 5, 6A/B, 7F, 9V, 14, 19A, 19F, 23F and 18C (included in sg18). The following NVTs were also detectable: 7C, 8F, 10A, 11A, 12F, 15A, 15B/C, 16F, 17F, 20, 22F, 31, 33F, 34, 35B, 35F and 38. Pneumococcal isolates that could not be typed using the primers stated in this study were designated as unknown serotypes.

### Statistical analysis

Statistical analysis was performed using the IBM SPSS Statistics 23. Descriptive statistics including frequencies were utilised. Median with interquartile range (IQR) was used for age and anthropometric indices, as these variables were not normally distributed.

Anthropometric indices were compared between the two groups (those who tested positive for pneumococcal carriage and those who tested negative) using the Mann–Whitney *U* test. The chi-square test was used to compare the frequency of HIV and immunisation status and other categorical variables between the two groups. Statistical significance was set at *p* < 0.05 for all tests.

### Ethical consideration

Ethical clearance was obtained from the Sefako Makgatho Health Sciences University Research Ethical Committee (SMUREC) prior to data collection (clearance number: SMUREC/M/47/2015/PG). Signed informed consent was obtained from the children’s parents or caregivers.

## Results

A total of 350 children were enrolled from December 2015 to April 2016, 44% (*n* = 153) of whom were female. The median age was 33.7 months (IQR of 16.3–69.5 months). The majority of the children (*n* = 242, 69.1%) were under 5 years of age.

The median length or height was 89 cm (IQR, 74.5 cm – 111 cm) and the median weight was 12.7 kg (IQR: 9.4 kg – 18.3 kg). The median HAZ was −0.97 (IQR: −2.08 to 0.0), while that for weight-for-age was −1.02 (IQR, −1.82 to −0.48). The median WHZ was −0.17 (IQR: −1.17 to 0.78).

On assessment of the immunisation status, 150 (43%) were fully immunised, 48 (18%) were partially immunised, 66 (19%) were not immunised and 86 (24%) had undetermined immunisation status. There were 256 children who were either 10 months and older or were born after December 2009 (i.e. after the introduction of PCV). The immunisation status of these 256 children is shown in Table 1.

**TABLE 1:** Immunisation status of children born after pneumococcal conjugate vaccine introduction and who were 10 months or older.

Immunisation status	Frequency	Proportion (%)
Fully immunised	148	58
Partially immunised	74	29
Not immunised	26	10
No RTHC (unknown status)	8	3

**Total**	**256**	**100**

RTHC, Road-to-Health card.

In terms of diagnosis, 74 (21%) of the children had pneumococcal-related conditions. Among these, pneumonia was the most common condition, with 60 (17%) children out of the 350 enrolled having this diagnosis. There were eight children with tonsillitis, five had meningitis and one had otitis media. The remaining 276 children had conditions not deemed to be pneumococcal-related. Among children with pneumococcal-related diagnoses, 42% (25/60) of those with pneumonia were fully immunised, as were 60% (3/5) of those with meningitis and 63% (5/8) of those with tonsillitis. The child with otitis media was fully immunised against pneumococcus.

Pneumococcus was isolated in nasopharyngeal swabs in 13% (*n* = 46). Children who were pneumococcus-positive were significantly younger (24.5 months; IQR: 9.5–48.1 months) than those who were pneumococcus-negative (34.8 months; IQR: 17.4–71.8 months), Mann–Whitney *U* test, *p* = 0.007. Sixteen (35%) of the 46 children with positive pneumococcal nasopharyngeal carriage were fully immunised, compared to 137 (45%) of the 304 who were pneumococcal carriage negative. There was no association between nasopharyngeal pneumococcal carriage and immunisation status among children eligible to have received a full course of PCV immunisation (*p* > 0.1). Eight (17%) of the children who were colonised with pneumococcus had pneumococcal-related conditions. Six of the eight had pneumonia and two had tonsillitis.

Anthropometric indices were similar in children who tested positive for pneumococcus colonisation and those who were pneumococcus-negative (Table 2).

**TABLE 2:** Age, gender, anthropometric indices and human immunodeficiency virus status of enrolled children in relation to their pneumococcal carriage.

Variable	Pneumococcal carriage									
	Positive (*n* = 46)				Negative (*n* = 304)			All children (*n* = 350)		
	Median	IQR	*n*	%	Median	IQR	%	Median	IQR	%
Age in months	24.5	9.5–48.1*		-	34.8	17.4–71.8	-	33.7	16.3–69.5	-
Females	-	-	24	52.1	129/304	-	42.4	153/350	-	43.7
WAZ	−1.45	−2.20 to −0.20	-	-	−0.93	−1.80 to −0.004	-	−1.02	−1.82 to +0.48	-
HAZ	−1.01	−2.16 to −0.02	-	-	−0.97	−2.07 to 0.00	-	−0.97	−2.08 to 0.00	-
WHZ	−0.57	−1.64 to +0.41	-	-	−0.11	−1.09 to +0.82	-	−0.17	−1.17 to +078	-
HIV-positive	-	-	6	13.0	27/304	-	8.9	33/350	-	9.4

WAZ, weight-for-age Z-score; WHZ, weight-for-height Z-score; HAZ, height-for-age Z-score; HIV, human immunodeficiency virus; IQR, interquartile range.

Note: Values for age, WAZ, HAZ and WAZ stated as median (interquartile range), except for gender and HIV-positive, which are stated as *n* (%).

* , Significantly lower than pneumococcal negative group, *p* < 0.05.

With regard to the HIV status, 307 (88%) were HIV-negative, 33 (9%) were HIV-positive and 10 (3%) had an unknown status. Forty (87%) children positive for pneumococcus colonisation were HIV-negative. Of the 304 children who were negative for pneumococcus on nasopharyngeal testing, 267 (87.8%) were HIV-negative, 27 (8.9%) were HIV-positive and 10 (3.3%) had an unknown HIV status (Table 3). There was no statistically significant association between HIV status and pneumococcal nasopharyngeal carriage (*p* > 0.1).

**TABLE 3:** Pneumococcal carriage results of study children by their human immunodeficiency virus status.

Pneumococcus	HIV-positive		HIV-negative		HIV unknown	
	*n*	%	*n*	%	*n*	%
Positive (*n* = 46)	6	13.0	40	87.0	0	0.0
Negative (*n* = 304)	267	87.8	27	8.9	10	3.3

HIV, human immunodeficiency virus.

**FIGURE 1:**
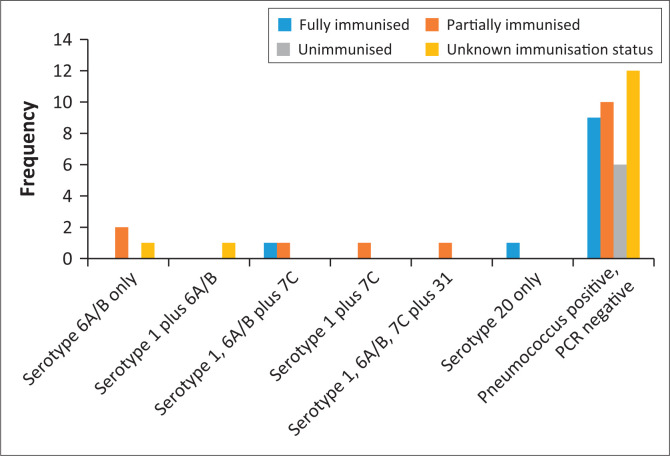
Pneumococcal serotypes identified among 46 children who had pneumococcal nasopharyngeal carriage.

Polymerase chain reaction testing for pneumococcal serotypes was conducted on all 46 pneumococcal-positive cases, and nine (20%) of them were positive for serotypes included in our PCR assays. Five serotypes were detected among these nine children: two vaccine serotypes (1 and 6A/B) and three NVTs (7C, 20 and 31). Four children had carriage of a single serotype: three with 6A/B and one with serotype 20. Five children had carriage of more than one serotype: one child had serotypes 1 and 6A/B, one child had serotypes 1 and 7C, two had serotypes 1, 6A/B and 7C and one had carriage of serotypes 1, 6A/B, 7C and 31. Serotype 6A/B was detected in seven (77.8%) of the nine children positive for pneumococcus on PCR testing. Serotype 7C was the commonest NVT identified (detected in four [44.4%] of the nine children). Figure 1 illustrates the pneumococcal serotypes among the 46 children whose nasopharyngeal swabs cultured pneumococcus. In the eight children with pneumococcal-related diagnoses and in whom nasopharyngeal swabs cultured pneumococcus, four were PCR-negative for the serotypes included in our assays. Of the four children with PCR-positive results, one had serotype 6A/B, one had serotypes 1 and 7C, one had serotypes 1, 6A/B and 7C and one had serotypes 1, 6A/B, 7C and 31.

Overall, 8 (2%) of the 350 children were colonised with vaccine serotypes, 5 (1%) carried NVTs and 37 (11%) carried pneumococci that could not be serotyped by the assays which we used. Of the eight children colonised with vaccine-serotype pneumococci, one was fully immunised, five were partially immunised and two had undetermined vaccination status.

Of the 256 children who were eligible to have been immunised with PCV, 32 (13%) tested positive for pneumococcus. Of the 150 fully immunised children, 143 (95%) were HIV-negative, 5 (3%) were HIV-positive and 2 (1%) had undetermined HIV status. There was no association between HIV and immunisation status (*p* > 0.1).

There was no statistically significant association between immunisation status and nasopharyngeal pneumococcal carriage (Table 4).

**TABLE 4:** Immunisation status and pneumococcal carriage of enrolled children.

Immunisations	Pneumococcal carriage				Total	
	Positive		Negative			
	*n*	%	*n*	%	*n*	%
Fully immunised	16	34.8	137	45.1	153	43.7
Partially immunised	14	30.4	31	10.2	45	12.9
Not immunised	2	4.3	64	21.1	66	18.9
No RTHC (unknown status)	14	30.4	72	23.4	86	24.6

**Total**	**46**	**100**	**304**	**100**	**350**	**100**

RTHC, Road-to-Health card.

## Discussion

In this study, we assessed the nasopharyngeal pneumococcal carriage prevalence in children attending an academic hospital in Pretoria approximately 7 years after the introduction of PCV, and we documented a carriage rate of 13%. This rate is lower than that noted in a South African trial conducted prior to the introduction of PCV, where the carriage rate was found to be 54% among immunised children.^24^ In the Gambia, a carriage rate of 35% was noted,^25^ while in Kenya the carriage rate was 17%.^20^ The rate which we observed is similar to that noted in the Kenyan study. This rate of 13% is, however, considerably lower than the carriage rate of 47% which was observed in a South African study conducted 2 years after the roll-out of PCV.^7^

Vaccine serotype colonisation was found to be 2% in our study, which is lower than the 22% (in children 12 years and younger) found in a South African study conducted 2 years after the PCV roll-out;^7^ this is likely an effect of immunisation. It is possible that the decline in vaccine-type colonisation has continued; however, it should be noted that Amies transport medium was used in our study rather than skim milk–tryptone–glucose–glycerine (STGG), which is the preferred transport medium for pneumococcus.^23^ It is possible that using STGG may have improved the viability of the organism and thus resulted in a different colonisation rate.

Pneumococcus nasopharyngeal carriage was more likely to be present in younger children in our study, which is in line with findings from other studies. A South African study assessing pneumococcal colonisation noted a prevalence of 73% in children younger than 5 years and 50% in the 6–12 year age group.^7^ In the Gambia, pneumococcal carriage decreased with increasing age, with the highest prevalence observed in younger children.^25^ A systematic review on pneumococcal carriage in sub-Saharan Africa also confirmed that pneumococcal carriage was highest for children less than 5 years and decreased with age.^26^ On the other hand, in an Italian study, pneumococcal colonisation was found to rise with increasing age.^27^

The HIV prevalence in our cohort was 9.4%, which is higher than the estimated vertical transmission rate of HIV from mother-to-child (< 5%) in South Africa.^28^ Similarly, another study had also shown a low mother-to-child transmission (MTCT) rate of 2.7%.^29^ However, in our study, the high prevalence rate was probably related to the fact that the children were enrolled from a hospital setting rather than from the general population. There was no correlation between HIV status and pneumococcal carriage. This was also corroborated in a study conducted in South Africa where no difference was found in carriage of pneumococcus in HIV-uninfected and HIV-infected children.^30^ In another South African study, however, the prevalence of pneumococcal colonisation in HIV-infected children was higher than those who were HIV-uninfected.^31^

In this study, only 43% of all enrolled children were fully immunised against pneumococcus. It is, however, important to note that some of the children were younger than 9 months; however, even among children who were 10 months or older and who were born after the introduction of PCV, 58% were fully immunised. This immunisation coverage is much lower than the government target of 95% coverage for immunisations by 1 year.^32^ It was also lower than the suboptimal coverage found in previous South African studies, where one study recorded 75% immunisation coverage at 9 months when all PCV vaccinations are completed and another study recorded an immunisation coverage of 72% at 9 months.^33,34^ In one of the studies, vaccine stock-outs were mentioned as a major contributor to the low immunisation coverage.^32^ However, as our study was conducted in children who were in a hospital, the children were likely to be under-vaccinated, as a lack of vaccination is a risk for hospitalisation with pneumococcal-related conditions.

In our study, no correlation was found between pneumococcal carriage and immunisation as the prevalence of pneumococcal colonisation was similar between children who were fully immunised and those who were partially immunised or not immunised. This has also been shown in other studies.^27^ In the Gambia, it was found that the overall prevalence of pneumococci was similar in fully immunised and partially immunised villages, with a trend towards lower prevalence among children (5–15 years age group) in fully immunised communities.^25^

In our study, the most common vaccine serotype was 6A/B, followed by serotype 1. Serotypes 6A and 6B could not be separated due to a lack of equipment to do pyrosequencing. Nonetheless, most pneumococcal isolates (80%) were negative for the serotypes included in our PCR assays. It should be noted that we tested for approximately one-third of the known serotypes, with adequate positive and negative controls built into the assay. We, however, only performed PCR testing on pneumococcal culture-positive nasopharyngeal swabs. Similarly, we could not test for all NVTs because of financial limitations.

Serotype 6A/B is commonly reported in studies assessing nasopharyngeal colonisation, including surveys from South Africa, Kenya, and a meta-analysis of studies conducted in low and lower middle-income countries.^7,20,35^ None of the samples were positive for serotype 15B, which was the commonest NVT causing IPD prior to the introduction of PCV.^36^ Nonetheless, the majority of pneumococcal isolates were not serotyped by our PCR assays. These were probably NVTs that were not included in the assays. The preponderance of these isolates is suggestive of serotype replacement.

We consider our inability to define the serotypes of the majority of the pneumococcal isolates obtained in our study, together with the use of Amies transport medium (rather than STGG) to be important limitations of the study.

The introduction of PCV and maintenance of high levels of vaccine coverage are expected to lead to a decrease in vaccine serotype colonisation and possibly achieve herd protection. While our study suggested a lower than previously reported prevalence of pneumococcal nasopharyngeal carriage in South African children, the stated limitations may have confounded the results.

Our findings indicate that serotype replacement colonisation is likely to have taken place. However, this needs to be confirmed in subsequent studies that are designed to enhance the yield and serotyping of pneumococcus.
